# The synergistic inhibitory effect of somatostatin-doxorubicin co-treatment on gallbladder carcinoma

**DOI:** 10.1186/1471-2407-7-125

**Published:** 2007-07-08

**Authors:** Ji-Yu Li, Zhi-Wei Quan, Qiang Zhang, Jian-Wen Liu

**Affiliations:** 1 Department of General Surgery, Xinhua Hospital, School of Medicine, Shanghai Jiao Tong University, Shanghai 200092, P.R. China; 2 State Key Laboratory of Bioreactor Engineering, New World Institute of Biotechnology, East China University of Science and Technology, Shanghai, P.R. China; 3 State Key Laboratory of Bioreactor Engineering & School of pharmacy, East China University of Science and Technology, Shanghai 200237, PR China

## Abstract

**Background:**

Gallbladder cancer is the most common billiary tract malignancy and carries a very poor prognosis. Somatostatin was recently shown to play an important role in the development of various tumors. In the current study, we evaluated the effect of doxorubicin on the chemosensitivity of gallbladder cancer cells and xenograft growth after treatment with somatostatin.

**Methods:**

Twenty-four hours after somatostatin treatment, doxorubicin was gradually added and the growth curve of gallbladder cancer cells was determined. Exponential-phase gallbladder cancer cells were treated with doxorubicine or co-treated with doxorubicine and somastatine and the respective IC_50 _values were determined. In addition, the inhibitory effect on the growth of gallbladder cancer xenograft on nude mice was evaluated using the same treatments as those described above.

**Results:**

Treatment of gallbladder cancer cells with somatostatin led to a block in the cell cycle at the S phase. Growth inhibition of gallbladder cancer cells by doxorubicin was concentration-dependent (*P *< 0.05). However, upon co-treatment with doxorubicin and somatostatin, the IC_50 _value significantly decreased as compared to that of cells treated with doxorubicine alone (*P *< 0.05). Interestingly, treatment with either doxorubicin or somatostatin did not significantly inhibit xenograft growth on nude mice, in contrast to a co-treatment with both drugs (*P *< 0.05).

**Conclusion:**

Somatostatin most likely sensitizes the chemotherapeutic effect and diminishes the cytotoxicity of doxorubicin in a gallbladder cancer cell line and in mouse gallbladder cancer xenografts.

## Background

Gallbladder cancer is the most common billiary tract malignancy and known as an aggressive and highly fatal disease with still a poor prognosis. Although relatively uncommon in Europe and the United States, gallbladder carcinoma is often reported in countries such as South-America, India, and Poland [1]. Symptoms often associated with gallbladder carcinoma include pain in the right hypochondrium, weight loss, anorexia, nausea and vomiting, and lump in the right hypochondrium [1]. In some rare cases, patients exhibit a primary non-Hodgkin lymphoma of the gallbladder [2]. Although the correlation between gallstones and gallbladder cancer is yet not well understood, the presence of gallstones is generally considered as an important risk factor for gallbladder cancer. Gallbladder carcinoma is most often reported in females and the 5-year survival rate varies between 0 and 10%. A recent long-term study reports that the survival rate of patients with gallbladder cancer has slightly improved over the past 85 years, but remains remarkably low [3]. This was also noticed in the P.R. China where the incidence rate of gallbladder cancer increased during the past decades whereas the survival rate remained unsatisfactory.

Despite recent research on therapeutic strategies against gallbladder carcinoma, surgical resection still appears to be the only potentially curative approach for this disease. Unfortunately, only a minority of patients is eligible to undergo surgery and, moreover, surgical removal of the gallbladder tumor does not necessarily implicate that the patient will recover in the long run [1]. Alternative therapies, such as radio- and chemotherapy, which has a substantial effect on metastatic pancreatic neuroendocrine tumors [4], for the treatment of gallbladder carcinoma have met with various degrees of success as gallbladder cancer cells are little sensitive to either of those approaches [1,5]. In a recent report, a clinical study on 5 patients diagnosed with advanced gallbladder carcinoma demonstrated that treatment with the pyrimidine antimetabolite gemcitabine might be a safe and effective treatment for advanced or metastatic gallbladder carcinoma [6]. The response of gemcitabine was enhanced in the presence of cisplatin [1]. Reports on the use of radiotherapy to treat gallbladder carcinoma are not encouraging [1]. Nevertheless, because the recurrence pattern is distant and local, it might be a helpful therapy to perform prior to or in combination with subsequent surgical resection as it appears to substantially enhance the chance for successful surgical resection [7]. In any case, it is obvious that future studies should focus on the development of successful strategies to treat gallbladder carcinoma.

Recent evidence showed that mis-regulation of the cell cycle may initiate tumor development [8]. Components involved in the proper regulation and functioning of the cell cycle are often altered in human cancer cells. A deregulation of crucial regulatory elements of the cell cycle may lead to an unlimited and uncontrolled mitosis and thereby provide selective cells with an advantaged growth pattern leading to the formation of cancer cells and thus the development of a tumor [9]. Consequently, several anticancer agents under study exhibit an inhibitory effect on the cancer cell's cell cycle.

Somatostatin (SST), a neuropeptide produced by neuroendocrine, inflammatory, and immune cells upon triggering different responses, has been used as a regulator of many hormones in clinical studies and plays an important role in the development of various tumors [10]. In the current study, the effect of SST on the regulation of growth and cell cycle of gallbladder cancer cells was investigated. The modulation effect of SST on the chemosensitivity of an anti-neoplastic drug, doxorubicin (DOX), which is one of the most effective anthracycline antibiotics with a broad antitumor spectrum [11], was evaluated as well. The ability of SST to enhance the cytotoxic effect of DOX on gallbladder cancer cells [12] was further elaborated as well as the effect of a DOX-SST co-treatment of nude mice harboring a gallbladder cancer xenograft.

## Methods

### Cell line culture and establishment of xenografts in nude mice

The human gallbladder carcinoma cell line GBC-SD was purchased from the Cell Bank of the Chinese Academic of Sciences (Shanghai). Cells were grown at 37°C in a humidified atmosphere containing 5% CO_2 _in RPMI 1640 medium (Gibco BRL, Carlsbad, CA) supplemented with fetal bovine serum (10%; Gibco BRL), glutamine (2 mM), penicillin (100 IU/ml), and streptomycin (100 μg/ml). Cells used in drug treatments or to inject mice were grown until the exponential growth phase.

Exponential-phase cells were trypsinized, washed with RPMI 1640, and suspended in saline solution to obtain a concentration of 5 × 10^6 ^cells/0.1 ml. Subsequently, 0.1 ml cell suspension was injected into each nude mouse (Experimental Animal Center, Chinese Academic of Science, Shanghai), subcutaneous in the axilla bilaterally. Formation of a tumor with a diameter of 5 mm was observed approximately 1 week after cell inoculation.

### Cell cycle analysis

GBC-SD cells were plated in six-well plates (Corning, New York, NY). Cells floating in the medium were combined with the adherent cell layer, which was then trypsinized. Cells were treated with SST (75 μg/ml; Serono Co., Switzerland) for 12, 24, 72, and 120 h and subsequently, 5 × 10^5 ^cells were washed, pelleted, and then incubated with 2 mg/ml RNase A in phosphate-buffered saline (PBS, 200 μl) and 0.1 mg/ml propidium iodide in 0.6% nonidet P-40 in PBS (200 μl) on ice for 30 min. Samples were immediately analyzed by flow cytometry using a fluorescence acquired cell sorter (FACS Calibur, Becton Dickinson Co., Franklin Lakes, NJ). The DNA content histograms were analyzed to determine the cell cycle phase distribution of the gallbladder cancer cells using the CELLQUEST software (Becton Dickinson).

### Cell viability assay

Cells from monolayer-grown GBC-SD cultures were harvested and dispensed in 96-well culture plates in 200 μl medium at a concentration of 10^5 ^cells per well. Cells were divided into three groups: DOX-use-only group, co-use-group (co-treatment with DOX (Sigma Chemicals, St.-Louis, MO) and SST), and control group. In the control group, PBS was added to the cells. DOX was applied in the following gradient concentrations: 0, 3.33, 6.66, 13.32, 19.98, and 33.30 μg/ml and the intracellular uptake was determined [11]. In the co-use group, cells were first treated with 75 μg/ml SST for 24 h followed by the addition of DOX in the gradient concentrations mentioned above. The cell viability was measured after 48 h of incubation using the 3-(4,5-dimethylthiazol-2-yl)-2,5-diphenyltetrazolium bromide (MTT) colorimetric assay [13,14]. Ten microliter MTT (5 mg/ml in PBS) was added to each well. After 4 h incubation at 37°C, supernatants were removed and replaced by 150 μl dimethyl sulfoxide. After formazan solubilization, the optical density at 590 nm (OD_590_) of each well was measured using an automated microplate reader (Bio-Rad Model 550, Microplate Reader, Hercules, CA). The cell viability was calculated using the following equation: cell viability = mean OD of one group/mean of the control × 100%

### Xenograft growth inhibition assay

Twenty-five nude mice bearing a xenograft were randomly divided into five groups: DOX-use-only group (2 mg/kg DOX), SST-use-only group (1 mg/kg SST), co-use group 1 (2 mg/kg DOX plus 1 mg/kg SST), co-use group 2 (2 mg/kg DOX plus 2 mg/kg SST), and the control group. The use and care of animals follwed the guidelines of the Ethical Committee of Xinhua Hospital, School of Medicine, Shanghai Jiaotong University. SST was injected daily, intraperitoneal, from the first day until the ninth day and DOX was injected every other day, intraperitoneal, from the second day until the eighth day (4 times). Those injections were combined in case of the co-use-group. Twenty-four days after the injection of mice with gallbladder cancer cells, tumors were harvested and the wet weight was determined.

### Statistical analyses

Because of the limited number of samples, the Wilcoxson rank sum test, a non-parametric method that allows to test independent samples, was applied to determine whether a given mean was significantly different from a respective control (*P *< 0.05). The SPSS software for Windows (version 13.0) was utilized to perform all the statistical analyses and the significance level (αvalue) was set at 0.05.

## Results

### Treatment of gallbladder cancer cells with SST causes cell cycle arrest

Because of the reported possible interactions between cell cycle deregulation and tumor development [9], we investigated the effect of SST on the cell cycle using the gallbladder cancer cell line GBC-SD grown to the exponential growth phase. This cell line exhibited a doubling time in the range of 25 to 30 h (date not shown). SST was applied in a concentration of 75 μg/ml and the ratio of cells in the S phase was determined after 12, 24, 72, and 120 h incubation.

**Figure 1:**
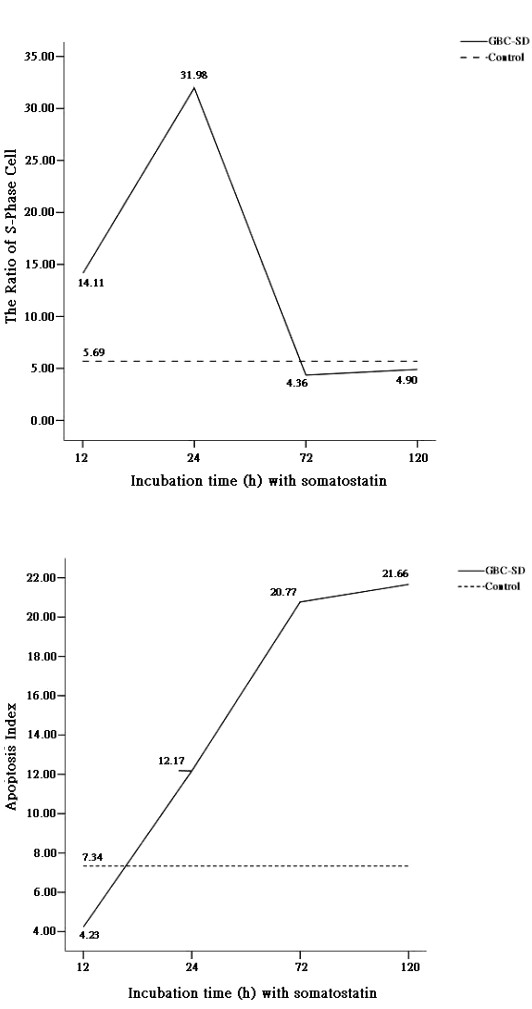
S-phase cell ratio (**A**) and apoptosis index (**B**) of gallbladder cancer cells (cell line GBC-SD) treated with SST (75 μg/ml) for 12, 24, 72, and 120 h. Means that are significantly different from the control group are indicated with an asterisk (*P *< 0.05).

The ratio of cells in the S phase increased 12 h after treatment with SST and was significantly higher than that for the control group 24 h after SST treatment (Figure 1A). However, the ratio of cells in the S phase rapidly decreased 72 h after SST treatment and was similar to that in the control group after 120 h incubation in the presence of SST (Figure 1A).

Simultaneously, for each time point at which the ratio of cells in the S phase was determined (Figure 1A), we measured the apoptosis index, indicative for the number of cells that underwent cell death. We observed that after 12 h SST treatment, the apoptosis index significantly increased as compared to that for the control group and kept increasing in time (Figure 1B).

### DOX and SST exhibit a synergistic inhibitory effect on the growth of gallbladder cancer cells

**Figure 2:**
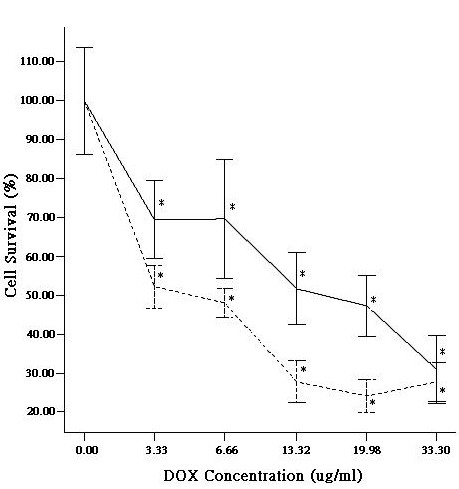
Inhibition of GBC-SD cell growth in the presence of DOX (solid line) or DOX and SST (dashed line). The relative survival percentage is shown. Means that are significantly different from the control group are indicated with an asterisk (*P *< 0.05).

In first instance, we determined the effect of DOX on cell growth using the GBC-SD cell line grown to the exponential growth phase. The concentration of DOX was gradually increased from 0 to 33.30 μg/ml (Methods) and the cell viability was measured in time. The cell growth was significantly inhibited at higher DOX concentrations (Figure 2). The DOX-mediated inhibition of gallbladder cancer cell growth was concentration-dependent (Figure 2).

Furthermore, we determined the effect of co-treatment with DOX and SST on the growth of GBC-SD cells (Figure 2). Cell growth inhibition occurred more rapidly in time as compared to that of cells treated with only DOX (Figure 2). The hastened cell growth inhibition was significant and confirmed by the calculation of the IC_50 _values which were 15.00 μg/ml DOX upon treatment of the gallbladder cancer cell line with only DOX and 4.50 μg/ml upon co-treatment with DOX and SST (Figure 2; *P *< 0.01).

### Inhibition of xenograft growth by co-treatment with DOX and SST

To investigate the effect of co-treatment with DOX and SST on the tumor growth in a mouse model system, we injected nude mice with gallbladder cancer cells grown to the exponential growth phase. Mice were either not treated, treated with DOX or SST, or co-treated with DOX and SST using two different concentrations of SST. Twenty-four days after the injection of mice with gallbladder cancer cells, the tumors were harvested and their wet weight was measured.

**Figure 3:**
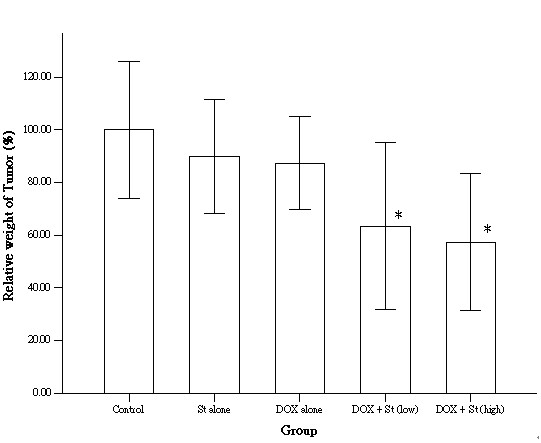
Inhibition of gallbladder cancer xenograft growth on nude mice upon treatment with SST (1 mg/kg), DOX (2 mg/kg), or a combination of both (2 mg/kg DOX with either 1 mg/kg or 2 mg/kg SST). Means that are significantly different from the control group are indicated with an asterisk (*P *< 0.05).

We observed that treatment with either DOX or SST alone did not significantly inhibit tumor growth in contrast to co-treatment with DOX and the lower concentration (1 mg/kg) of SST (Figure 3). The latter growth inhibition, indicated by a lower wet weight of the tumors, was significant (*P *< 0.05). Because the number of mice tested in this experiment was relatively low, we evaluated and calculated the statistical power. Using the nQuery Advisor^® ^software to perform the Wilcoxson rank sum test, we determined the power for the two significant tests in Figure 3, examining the difference in the average tumor weight between mice co-treated with 2 mg/kg DOX and 1 mg/kg SST and control mice, and mice co-treated with 2 mg/kg DOX and 2 mg/kg SST and control mice. The power was 0.51 and 0.64, respectively.

## Discussion

Gallbladder carcinoma is a devastating disease for which the only feasible cure, at least for some patients, is surgical resection. Unfortunately, surgical resection only helps some gallbladder cancer patients and the long-term survival rate remains low [1]. It is remarkable that to date no successful alternative therapies have been developed, mainly because gallbladder cancer cells are relatively little sensitive to commonly used therapies such as radiotherapy and chemotherapy. Therefore, it is crucial to engineer and design new strategies that may ultimately lead to the discovery of an effective and safe, non-surgical treatment of gallbladder carcinoma.

More evidence is becoming available that demonstrates the interconnection between cell cycle deregulation and tumor development [9]. It has been demonstrated that a positive correlation exists between the status of p53, a tumor suppressor protein that in response to cellular stimuli controls the expression of genes involved in the control of cell cycle arrest and cell death [15], and the chemosensitivity of, for instance, ovarian cancer cells [16,17]. In fact, environmental factors that cause DNA damage lead to the phosphorylation of p53 which prevents a pathway required for the transcription of S-phase genes, thereby maintaining G1/S phase arrest [18]. Interestingly, SST induced p53 production in human breast cancer cells, exhibiting p53 depletion, which resulted in cell growth arrest [19]. It has been postulated that SST directly stimulates tumor cell apoptosis via SST receptor 3-dependent G-protein signaling which causes, among other factors, the induction of p53 suppressor gene expression [20]. Here, we demonstrate using flow cytometry that the ratio of gallbladder cancer cells in the S phase increases upon treatment with SST for 24 h. While this remains to be proven, those observations strongly suggest that gallbladder cancer cells treated with SST are arrested at G1/S transition, a process which is likely mediated by p53.

SST has been used as a regulator of secretion of many gastrointestinal hormones in the clinic for a long time. In addition, SST and its stable analogues were shown to exert an anti-proliferative effect on various cancerous cells both in vitro and in vivo [10]. This is confirmed by our results as we demonstrated that SST significantly inhibits cell growth in a gallbladder cancer cell line in a concentration-dependent manner [21]. Thus, SST likely inhibits gallbladder cancer cell growth by arresting the cell cycle at the S phase and inducing apoptosis which is the subject of further studies. The latter observations illustrate that gallbladder cancer cells appear to be relatively sensitive to treatment with SST which may have important clinical applications.

However, the effect of SST on the growth of gallbladder cancer cells was observed at fairly high SST concentration (i.e., 75 μg/ml) which is tenfold higher than the SST concentration generally used in other applications. This high concentration would prohibit its use in human patients as it might have severe cytostatic and cytotoxic effects [9]. In addition, we noticed that such a high SST concentration did not affect the growth of gallbladder cancer xenografts on nude mice. Therefore, we investigated the hypothesis whether co-treatment with a chemotherapeutic drug that functions by interrupting DNA synthesis in the S phase of the cell cycle would enhance the inhibitory effect of SST on gallbladder cancer cell growth and thus lowers the workable SST concentration.

DOX is an anthracycline antibiotic often used as a classic chemotherapeutic drug which exhibits an antitumor effect caused by intercalation into DNA molecules [11]. Unfortunately, gallbladder cancer cells appear to be rather resistant to treatment with DOX and many other chemotherapeutic agents. Although gallbladder cancer cell growth inhibition by DOX was observed and concentration-dependent [22], the DOX concentration needed to induce antitumor effects was fairly high. Strikingly, a co-treatment of gallbladder cancer cells with SST and DOX reduced the IC_50 _value from 15 μg/ml DOX in the presence of DOX alone to 4.5 μg/ml in the presence of both DOX and SST. This co-treatment, in contrast to treatments with either DOX or SST, also significantly reduced the wet weight of gallbladder cancer xenografts developed on nude mice.

Taken together, these data strongly suggest that modulation of the cell cycle by treatment of SST could sensitize chemotherapy, both in a gallbladder cancer cell line and gallbladder cancer xenografts developed on nude mice. Although the underlying molecular mechanisms that could explain this synergistic inhibitory effect on gallbladder cancer cell growth remain to be elucidated, it is possible that treatment with SST reduces the expression of high affinity SST receptors in gallbladder cancer cells, an effect of SST that has been demonstrated in pancreatic tumor cell lines [23], which may alter the landscape of receptors and other cell surface proteins on gallbladder cancer cells. The latter may substantially increase the sensitivity of those cancer cells to DNA synthesis-disrupting chemotherapeutic agents. Future work is required to further evaluate this hypothesis.

Although the way to a successful non-surgical therapy for gallbladder carcinoma is still long, the results presented herein provide new insights in a possible chemotherapeutic approach in combination with an SST treatment which would allow gallbladder cancer cells to arrest their cell cycle. Future studies are required to unravel the underlying molecular pathways that explain the synergistic inhibitory effect of the DOX-SST co-treatment on gallbladder carcinoma development and to pinpoint the selectivity of those drugs in human patients diagnosed with advanced gallbladder carcinoma.

## Conclusion

Pre-treatment of a gallbladder carcinoma cell line with somatostatin increased sensitivity to doxorubicin, as evidenced by inhibition of cancer cell growth both *in vitro *and *in vivo*.

## Abbreviations

3-(4,5-dimethylthiazol-2-yl)-2,5-diphenyltetrtazolium bromide, MMT; doxorubicin, DOX; human gallbladder carcinoma cell line, GBC-SD; somatostatin, SST

## Competing interests

The author(s) declare that they have no competing interests.

## Authors' contributions

All authors read and approved the final manuscript.

JY LI contributed to the experimental design, in vitro cell culture and MTT, and FCM experiments.

ZW Quan contributed to the animal model experiments.

Q Zhang contributed to the in vivo experiments.

JW Liu contributed to the experimental consult.

## Pre-publication history

The pre-publication history for this paper can be accessed here:


